# Household air pollution and its effects on health

**DOI:** 10.12688/f1000research.7552.1

**Published:** 2016-10-28

**Authors:** Komalkirti Apte, Sundeep Salvi

**Affiliations:** 1 Chest Research Foundation, Kalyaninagar, Pune, India

**Keywords:** air quality, particulate matter, air pollutants

## Abstract

Household air pollution is a leading cause of disability-adjusted life years in Southeast Asia and the third leading cause of disability-adjusted life years globally. There are at least sixty sources of household air pollution, and these vary from country to country. Indoor tobacco smoking, construction material used in building houses, fuel used for cooking, heating and lighting, use of incense and various forms of mosquito repellents, use of pesticides and chemicals used for cleaning at home, and use of artificial fragrances are some of the various sources that contribute to household air pollution.

Household air pollution affects all stages of life with multi-systemic health effects, and its effects are evident right from pre-conception to old age. *In utero* exposure to household air pollutants has been shown to have health effects which resonate over the entire lifetime. Exposures to indoor air pollutants in early childhood also tend to have repercussions throughout life. The respiratory system bears the maximum brunt, but effects on the cardiovascular system, endocrine system, and nervous system are largely underplayed. Household air pollutants have also been implicated in the development of various types of cancers.

Identifying household air pollutants and their health implications helps us prepare for various health-related issues. However, the real challenge is adopting changes to reduce the health effects of household air pollution and designing innovative interventions to minimize the risk of further exposure.

This review is an attempt to understand the various sources of household air pollution, the effects on health, and strategies to deal with this emergent risk factor of global mortality and morbidity.

## Introduction

In the words of Rosalynn Carter, “There is nothing more important than a good, safe and secure home”. It is believed that a house is the most secure and healthy environment for any individual. However, the house can also be a source of various air pollutants that can have a significant adverse impact on health.

**Figure 1.**
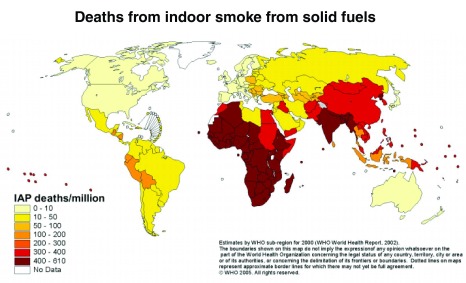
Global indoor air pollution (IAP) mortality per million population. Reprinted with permission from WHO website: http://www.who.int/heli/risks/indoorair/en/iapmap.pdf (accessed on 12th Oct 2016).

According to the latest World Health Organization (WHO) report, 8 million people die every year globally because of air pollution ^1^. Among these, 4.3 million die because of air pollution from household sources and 3.7 million die because of ambient air pollution ( Figure 1).

According to the Global Burden of Disease Report, household air pollution is the leading cause of disability-adjusted life years (DALYs) in Southeast Asia and the third leading cause of DALYs globally ^2^. Interestingly, ambient air pollution is the sixth leading cause of DALYs in the Southeast Asia region and the ninth leading cause of DALYs globally. Until recently, air pollution from motor vehicles and industries was perceived as a bigger threat to human health. It is only now that we have started appreciating the significant adverse impacts of household air pollution on human health.

This review is an attempt to understand the various sources of household air pollution, the effects on health, and strategies to deal with this emergent risk factor of global mortality and morbidity.

## Housing across the globe

### Houses in developed countries

Most developed countries have houses built on a concrete base with a wooden framework and asbestos sheets. Building material is further packed with fire-retardant material to reduce the fire hazards in such houses. Flooring usually is made of polished and varnished wood. Many houses also feature vinyl flooring or carpeted flooring. Clean liquefied petroleum gas (LPG), natural gas, or electricity is used for cooking. Many of these houses are fitted with air conditioners to maintain a comfortable room temperature. Many houses feature an electric furnace or a wood fireplace for heating, more so in areas prone to snowfall. To ensure effective thermal conditioning of the house, insulation material is used extensively. Upholstery such as heavy drapes, thick sofa covers, decorative throws, and pillows are a regular feature in these houses. Varnished and polished wood is used for furnishings. Very often, particle board material is used for furniture for its ease and economy of use. Bedding material used in these households includes down feathers, coir, and foam. The use of scented candles, room fresheners, potpourri, frankincense, and so on is a usual occurrence. In this era of advanced technology, most of these houses are equipped with modern gadgets such as printers, copiers, and fax machines. Improved personal hygiene also involves the extensive use of cleaning agents, perfumes, and deodorants, regular painting and varnishing, floor and furniture polishing, and so on. Insect repellents are used in many of these households in order to keep the home environment clean.

### Houses in developing countries and rural communities

**Figure 2.**
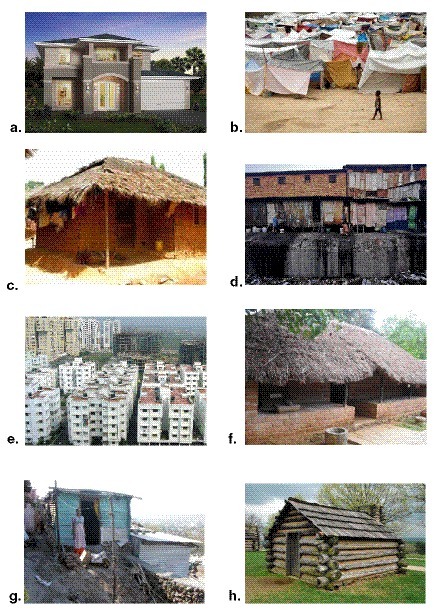
Types of housing. **a**. American house **b**. Temporary cloth shelters **c**. Mud brick shanty **d**. Urban slums in India **e**. Urban apartment complex in India **f**. Brick and wood house with a concrete floor **g**. Tin shanty **h**. Wooden log cabin.

Most developing countries, on the other hand, use stones, bricks, concrete, and cement to build their houses. Although the use of air conditioning is on the rise in warmer developing countries, most houses rely on natural ventilation through open windows and shutters. Overcrowding and increased industrialization have led to housing within close vicinity of industries and heavy traffic-dense roads. The poor socioeconomic strata of society in these countries continue to live in ill-ventilated and ill-lit houses made of bamboo, wood, crop residues, tin sheets, and sometimes cloth. Biomass for cooking, heating, and lighting is used widely in these parts of the world. Paints and whitewash are commonly used in these houses. Insect infestations remain a major problem in developing and rural houses, and repellents are widely used. The use of fragrances through air fresheners, frankincense, scented candles, and potpourri is a rising trend. Increasing demands for improved housing have led to a growth spurt of housing complexes with a faster turnover time period. This in turn has affected the quality of the finished housing, leading to poor quality of the construction material, faulty electrical and plumbing work, and an overall poor quality of housing ( Figure 2).

## Sources of household air pollution

Apart from the use of solid cooking fuels, there are more than 60 risk factors associated with the increased burden of household air pollution ^3^.

### Cooking-related household air pollution

One of the major sources of household air pollution, especially in developing countries, is fuel used for cooking as well as heating practices. Homes from developed countries and many houses in the developing world use electricity, natural gas, or clean LPG for cooking, whereas houses in rural communities and some houses of the developing world use biomass fuel for cooking.

Natural gas is primarily methane, whereas LPG is primarily propane or a mixture of propane and butane. Natural gas requires less air for combustion (an air-to-gas ratio of 10:1). LPG, on the other hand, requires more air for combustion (an air-to-gas ratio of 25:1), releasing almost three times the energy released by burning natural gas (93.2 MJ/m ^3^ through LPG versus 38.7 MJ/m ^3^ through natural gas). LPG is relatively denser than air, which is further denser than natural gas (1.52:1:0.55). Gas leakage when using LPG therefore tends to settle in the household air at human levels, whereas leakage of natural gas ascends toward the ceiling, reducing health effects. Burning of natural gas not only produces a variety of gases such as sulfur oxides, mercury compounds, and particulate matter but also leads to the production of nitrogen oxides, primarily nitrogen dioxide.

Biomass fuel includes wood, crop residue, animal dung cakes, and wood charcoal. Approximately, 3 billion people, or half the world’s population, use biomass for cooking or heating across the globe and burn about 2 million kilograms of biomass every day. China alone is responsible for 420,000 annual deaths due to indoor air pollution caused by the use of solid fuels ^4^. These homes have very high levels of particulate matter and gaseous air pollutants such as carbon particles, iron, lead, cadmium, silica, phenols and free radicals, carbon monoxide (CO), nitrogen dioxide, sulfur dioxide, formaldehyde, hydrocarbon complexes, and other inorganic and organic substances which include polycyclic aromatic hydrocarbons (PAHs), volatile organic compounds, and chlorinated dioxins. Various studies ^5^ have shown that, on average, the levels of particulate matter of less than 2.5 microns in mean aerodynamic diameter (PM _2.5_) in such houses range from 500 to 1,500 µg/m3, which is very high, as the permissible indoor level of PM _2.5_ according to the WHO ranges between 10 and 50 µg/m ^3^ in a 24-hour mean value ^6^. High levels of CO, especially during the burning of charcoal, are also produced. However, burning of wood produces the least amounts of PM _2.5_ and CO among the biomass fuels ^7^.

The method of cooking also has an impact on the levels of particulate matter released in the air. Stir frying, deep or shallow frying, charbroiling, roasting, and grilling have different emission levels of particulate matter. The type of meat, the amount of fat in the meat, and the type of oil used for cooking also determine the emission levels. Meat charbroiling, for example, emits large quantities of particulate matter of 0.1 to 0.2 µm in mean aerodynamic diameter ^8^. Meat frying and charbroiling contribute to about 21% of the particulate matter emitted. Regular meat emits about 40 g of particulate matter per kilogram of meat when charbroiled, whereas lean meat emits about 7 g of particulate matter per kilogram of meat when charbroiled ^8^. When subjected to frying, the same meat emits about 1 g of particulate matter per kilogram of meat. Oil used in cooking emits a significant amount of PAHs, which further add to the household air pollutants. Stir frying, a popular cooking style in Chinese cuisine, has been shown to emit particulate matter ^9^ ranging from 300 to 1,700 μg/m ^3^.

### Smoking

Smoking tobacco in any form within the confines of a house is a major source of household air pollution. Globally, there are approximately 1.1 billion smokers, a number which is steadily on the rise. Cigarette smoke contains 7,357 different chemical compounds such as benzene, CO, PAHs, heterocyclic amines, cyanide, formaldehyde, terpenoids, phenols, nicotine, and heavy metals. Burning of tobacco also emits considerable amounts of PM _2.5_ (burning one cigarette emits 7 to 23 mg of PM _2.5_) ^10^.

Tobacco smoke could be first-hand, second-hand, or third-hand smoke. A person who smokes within the confines of his house is exposed to the smoke himself (first-hand smoke). Other occupants of the house inhaling these fumes but not actually smoking are exposed to second-hand smoke. The particles emitted during smoking settle on the furnishings, hair, clothes, and the floor. These particles remain suspended in the household air for quite a while even after the primary smoker has exited the premises. This constitutes third-hand smoke.

### Temperature control-related household air pollution

Temperature control may involve both heating and cooling of the household environment. Houses in developed countries and some houses in developing countries use air conditioning for temperature and humidity control. To ensure effective conditioning of the house, insulation material and drafting control are used extensively. Preventing the temperature-controlled air from escaping the closed environment leads to poor ventilation in these houses and further causes accumulation of the particulate matter pollutants inside the house ^11^. Furthermore, inadequately cleaned air conditioning units are breeding grounds for various fungi and bacteria.

Opening windows is a form of natural ventilation. Ambient air pollutants containing copper, iron, potassium, nickel, silicon, vanadium, and zinc, with a particle size of between 2.5 and 10 microns, and ozone gas easily find their way into these houses through the natural ventilation, in turn contributing to household air pollution ^12^. In particular, houses that are close to traffic-heavy roads, which are populated with vehicles using diesel as fuel, tend to have a higher load of household air pollutants. Diesel exhaust particles can settle on the pollen of roadside trees and consequently find their way into an open household ^13^. Carbon particles, soot, diesel exhaust-laden pollen, and organic particulate matter are therefore found to be in very high concentrations in these houses which use natural ventilation.

Biomass has been used for heating in several parts of the world. Burning of biomass emits not only PM _2.5_ but also high levels of PM _10_ (particulate matter of less than 10 µm in mean aerodynamic diameter) up to 5,000 µg/m ^3^ (limits permissible by the WHO are 100 µg/m ^3^ as a daily average). Novakov and Corrigan showed through their study on thermal characterization of biomass smoke particles that black carbon is in fact a biomarker of biomass fuel combustion ^14^. Burning of charcoal also emits a substantial amount of polyaromatic hydrocarbons and particulate matters yet considerably lower than burning wood ^15^.

### Insecticides and pest control

**Figure 3.**
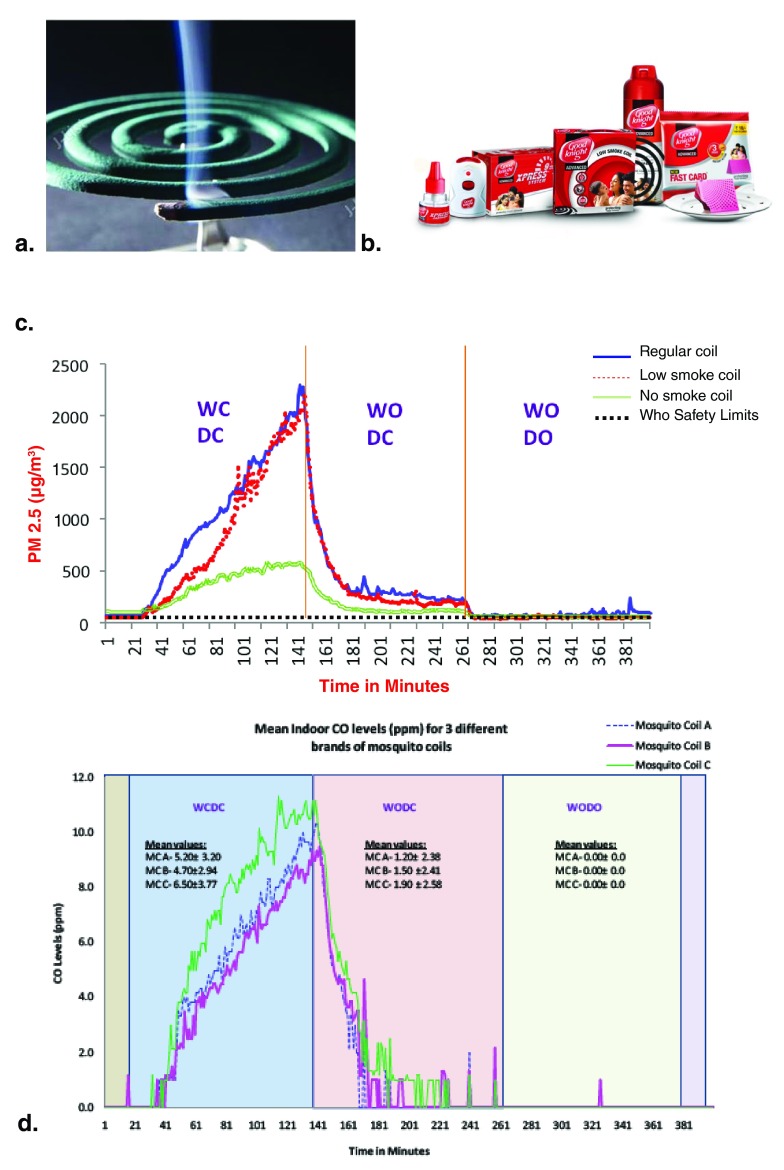
Mosquito repellents and the pollutant emission levels. **a**. Burning mosquito coil **b**. Range of mosquito repellents manufactured by Goodnight© **c**. PM _2.5_ levels when burning three different brands of mosquito coils with different modes of ventilation **d**. CO levels when burning three different brands of mosquito coils with different modes of ventilation. CO, carbon monoxide; DC, door closed; DO, door open; PM _2.5_, particulate matter of less than 2.5 microns in mean aerodynamic diameter; WC, window closed; WHO, World Health Organization; WO, window open.

The developing and rural world is still grappling with communicable and infectious diseases, usually borne by vectors such as mosquitoes, insects, and ticks. One of the biggest menaces is through mosquito-borne diseases such as malaria and dengue. In an effort to curb the growing mortality and morbidity rates of these mosquito-borne illnesses, mosquito control is a burning need. This is done by the use of chemical repellents. The most widely and commonly used repellent is the mosquito coil. Approximately 2 billion people across the globe use mosquito coils to ward off the dangers associated with mosquito-borne illnesses. A standard mosquito coil is made up of 0.1% of the active repellent pyrethroids, whereas the remaining 99.9% contains binders, resins, and flammable material such as coal dust and coconut husks. Twelve million such coils are sold every year in the world. The coil is ignited and allowed to smoulder for 6 to 7 hours. Ideally, the coil is meant to be lit and smouldered in a room with windows and doors closed to get the most desired effect. Previous studies have shown that burning of one such coil emits particulate matter equivalent to burning 100 cigarettes and PAH equivalent to burning 50 cigarettes. We have recently shown that levels of PM _2.5_ and CO are 2,200 times and 10 times the limits permissible by the WHO, respectively, when the mosquito coil is burnt with doors and windows closed. Improving ventilation by opening the windows but retaining closed doors reduced the pollutant levels markedly but remained at 500 times and twice the limits permissible by the WHO ^16^. Only when both the doors and the windows are kept open do the pollutant levels drop down to the safety limits. However, this defeats the use of the mosquito coil to control the mosquito menace. Other forms of mosquito repellents such as vaporizers, sprays, ointments, and medicated papers do not produce as much particulate matter but produce gaseous air pollutants that are irritants to the airway mucosa ( Figure 3).

In regions where malaria or mosquito-borne diseases are prevalent, household spraying with dichlorodiphenyltrichloroethane (DDT) or pyrethroids is very common. Repeated spraying of these chemical repellents leads to accumulation of these toxicants in the house. Interestingly, house dust concentrations serve as an excellent metric for estimating concentrations of insecticide exposure within the house. South African researchers showed a markedly high concentration of residual DDT in homes 2 months after spraying the insecticide (910 versus 1.3 μg/m ^2^ in homes not sprayed) ^17^. Similarly, a study from Mexico showed higher levels of residual insecticides as late as 3 years after spraying houses with DDT (30.8 μg/g of house dust versus 0.7 μg/g of house dust in homes not sprayed with the insecticide) ^18^.

### Perfumes, deodorants, and cleaning agents

Poorly ventilated homes tend to accumulate kitchen odours as well as concentrate the household air pollutants. This warrants the use of perfumes and scents to improve the level of hygiene in the house. Improved hygiene involves the use of cleaning agents, perfumes and deodorants, scented candles, and so on to make the house more cosy and comfortable. Steinemann ^19^ studied 37 commonly used consumer products, which include air fresheners, laundry products, personal care products, and cleaning agents, to identify the volatile organic compounds released by them. Interestingly, this study identified 156 different volatile organic compounds, of which the US Food and Drug Administration classified at least 42 as toxic or hazardous. Similar observations have been made by other researchers as well ^20^.

**Figure 4.**
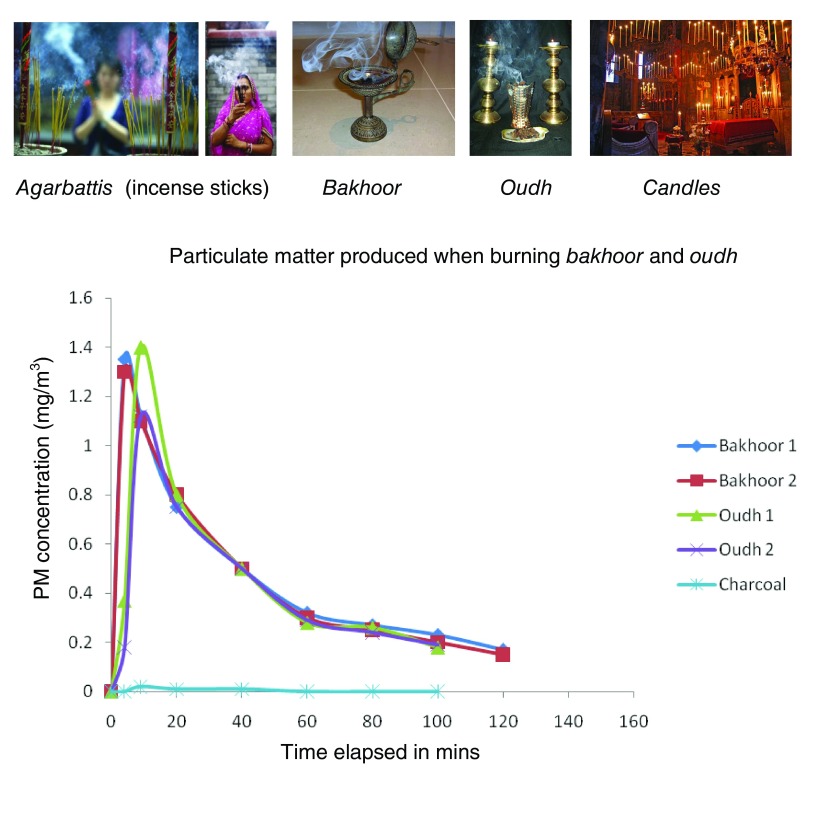
Fragrances used across the globe and their contribution to air pollution. Adapted from Cohen R *et al.* ^21^.

Almost all religions resort to using some form of fragrances in their everyday religious activities. Hindus and Buddhists burn incense sticks and fragrances, offer oil-lit lamps, and burn smoke sticks called *dhoop* during their daily prayer service. Hindu marriages often involve the burning of a sacred fire with wood and animal dung cakes for at least 2 to 3 hours per ceremony. There are 3 million places of religious worship in India alone, and 10 million marriages are performed there each year. Similarly, Christians burn candles during prayers and more so during Easter and Christmas. Islamic homes tend to use fragrances in the form of *Bakhoor* and *Oudh*, which emit fragrances when placed over hot charcoal. Various studies have reported that toxic levels of air pollutants are emitted when these fragrances are burnt. The incense sticks called *agarbattis*, made of finely ground fragrant material, are bound with binders, usually *makko*, around a supporting bamboo stick. India alone sells incense sticks worth 225 million USD per year and this has been growing annually at 10%. Among the Chinese, 76.9% currently burn incense at home every day and over 90% of the population has been using these for over 20 years. Burning of these fragrances emits high levels of PAHs, benzene, nitrous oxide, and CO ^21^ ( Figure 4). Similarly, burning of candles produces PM _2.5_ and PM _10_ to the tune of 1,200 and 200 µg/m ^3^, respectively ^22^.

### Building material

Paints and varnishes used in houses emit significant amounts of volatile organic compounds, increasing the burden of household air pollutants. Particulate board furniture is also responsible for emission similar to that of volatile organic compounds largely because they are held together by adhesives that emit volatile organic compounds. Insulation material used in buildings has also been implicated in the emission of volatile organic compounds ^23^. Asbestos used in sheets in the construction of houses leaves the finished houses with fine asbestos dust particles. Similarly, silicon particles are found in abundance in houses made of bricks and cement. Improvements in technology have led to digitalization in houses. Every household today has hi-tech electronic gadgets such as computers, tablets, iPads, printers, and mobile phones. These have been implicated in increased levels of ozone in the houses. Polybrominated diphenyl ethers (PDBEs) are used as fire retardants in foam-containing furniture and electronics. These emit PentaPDBEs and DecaPDBEs in minute quantities which contribute to household air pollutants ^24^.

Faulty plumbing, alone or coupled with weather conditions, has led to an increasing incidence of indoor wall dampness ^25^. These walls form an ideal environment for the growth of fungi such as Alternaria, Aspergillus, Cladosporium, and Penicillium ^26^. Along with fungi, damp mouldy walls are also a breeding ground for several species of Gram-positive and Gram-negative bacteria which include Streptococcus, Micrococcus, Staphylococcus, Mycobacterium, Nocardia, and Streptomyces ^27^. Budding spores of these fungi, microbial particulates, volatile organic compounds, mycotoxins, and endotoxins of the bacteria contribute a great deal to household air pollution ^28^.

### Other sources

Many household air pollutants, such as PM _2.5_, benzopyrene, lead, ozone, nitric oxide, sulfur dioxide, organophosphates in household pesticides, and tobacco smoke, have the potential to disrupt the endocrine system of humans as well as animals. These compounds have been termed “obesogens”, as they lead to metabolic syndrome and obesity ^29^.

Many pathogens such as bacterial droplets, viral droplets, and fungal spores remain suspended in the household air after an infection in the family. These droplets settle on household surfaces, acting as fomites for the spread of infection.

Table 1 illustrates the various sources of household air pollution.

**Table 1.** Sources of household air pollution

Sources of household air pollution	Examples
Cooking methods (using liquefied petroleum gas or electricity)	Stir frying, frying, roasting, grilling, baking, basting, and broiling methods which lead to an increase in particulate matter (PM _2.5_)
Biomass fuels	Wood, crop residue, animal dung, and charcoal
Tobacco smoke	Active smokers and second-hand and third-hand smoke
Incense sticks Mosquito repellents	Agarbatti and dhoop (incense sticks), *Bakhoor*, and *Oudh* Mosquito coils, flammable paper mats, and aerosols
Cleaning agents, products of personal care, air fresheners, wood varnishes, paint, and carpet solvents Fire-retardant foam-containing furniture, electronic gadgets, and building material	Volatile organic compounds Polybrominated diphenyl ethers
Fungi such as Aspergillus, Cladosporium, and Penicillium Bacteria such as Legionella	Damp walls and ill-maintained air conditioning
Domestic pets	Pet dander

PM _2.5_, particulate matter of less than 2.5 microns in mean aerodynamic diameter.

## Effects of household air pollution

**Figure 5.**
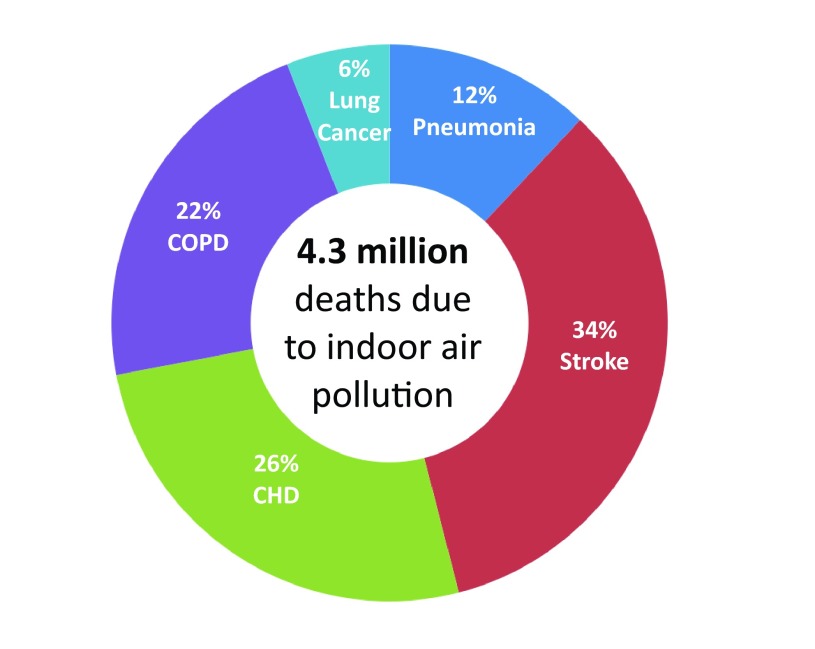
Deaths related to indoor air pollution. CHD, coronary heart disease; COPD, chronic obstructive pulmonary disease.

Of the 4.3 million people who die every year because of household air pollution, 60% die because of cardiovascular diseases and 40% die because of pulmonary diseases ^1^ ( Figure 5). Short-term effects of exposure to household air pollutants confer an increased risk for deaths because of cardiovascular as well as respiratory causes. A 10 µg/m ^3^ increase in indoor PM _10_ has been shown to increase cardiovascular mortality by 0.36% and respiratory mortality by 0.42%. Similarly, a 10 µg/m ^3^ increase in indoor PM _2.5_ has been shown to increase cardiovascular mortality by 0.63% and respiratory mortality by 0.75%. In the long term, every 10 µg/m ^3^ increase of household PM _10_ increases the risk of mortality by 23% to 67% ^30^.

Household air pollution begins to affect a human even during fetal life. Increased household air pollution increases oxidative stress, which has been implicated in decreased fertility or, in some cases, even infertility. Increased oxidative stress leads to decreased sperm motility and poor zygote quality. It also plays an important role in increasing insulin resistance, which is associated with polycystic ovarian disease, a major cause of infertility ^31, 32^. With improved technology in assisted reproduction, many infertile couples turn to *in vitro* fertilization for conception. The presence of carbon particles, through lack of a carbon filter, in the air of the chamber where the actual fertilization process is carried out leads to poor cleavage formations in a blastocyst, resulting in poor fertilization. Pregnant women exposed to fumes from the burning of biomass and solid fuel were reported to have a twofold risk of developing symptoms of pre-eclampsia or eclampsia as compared with those who used cleaner cooking fuels ^33^. Pre-eclampsia and eclampsia are known to have detrimental effects on the unborn baby because of a compromised umbilical cord supply during the pre-eclamptic or eclamptic symptoms. These include intra-uterine growth retardation, low birth weight, pre-term delivery, and poor lung maturation.

One may believe that a fetus is safe within the safest and purest confines of its mother’s uterus. However, a study of 10 newborn infants in New York by the Environmental Work Group revealed that these infants, born to mothers exposed to pollutants, had as many as 232 pollutants circulating in the cord blood collected at birth ^34^. A research group from Harvard University (Cambridge, MA, USA) reported that pregnant mothers with an increased exposure to PM _2.5_ and black carbon (as found in houses which use biomass for heating or cooking) in their third trimester delivered babies that had an increased systolic blood pressure ^35^. These findings, as the authors claim, need to be followed up longitudinally to understand whether this has any health implications in later adult life. Similarly, another study reported that increased exposure to polycyclic aromatic hydrocarbons and heavy metals (especially lead and mercury) in the second trimester of pregnancy resulted in decreased length of the baby at birth ^36^. They also have lower heights, which do not recover later in life. In fact, height charts of these children have been found to be in the lower percentiles. The effect of perinatal exposure to PAHs has also been studied, revealing compromised lung function in otherwise-healthy children ^37^. Various studies have shown that indoor air pollution is associated with a 38% increased risk of low birth weight and an alarming 51% increased risk of stillbirths ^38^. Exposure of pregnant mothers to indoor air pollution specifically by cooking on cook stoves has been shown to result in fetal thrombosis, which leads to an increased incidence of stillbirths and infant mortality.

Increased exposures to household air pollutants have been shown to be associated with an increased body mass index ^39^. This has been observed more in children born of mothers exposed to cigarette smoke as well as high levels of PM _2.5_ and PM _10_^40^. Children who tend to be within close vicinity of their mother while she goes about her domestic chores have been found to have increased exposures to trihalomethanes, volatile compounds commonly found in cleaning products ^41^. This exposure confers an increased risk of developing respiratory ailments such as allergic rhinitis, asthma, allergic conjunctivitis, eczema, atopic dermatitis, and recurrent chest infections.

It is interesting to note that exposure to household air pollutants is closely associated with the risks of developing respiratory infections, mainly pneumonias and tuberculosis ^42^. The WHO report of 2014 has revealed that approximately 120 million children, under the age of 5 years, have pneumonias and that, of these, 8 million require hospitalization and 1.1 million succumb to the pneumonia ^1^. Alarmingly, over 50% of these pneumonia deaths are attributed to household air pollution due to the burning of biomass. The burning of biomass fuel or any other fossil fuel increases the concentration of black carbon in the air. Black carbon in the ambient air is due largely to vehicular emissions, whereas black carbon in the household air is by and large a result of the combustion of biomass fuels ^43^. An increased amount of inhaled black carbon leads to an increased susceptibility for infections and decreased immunity ^44^. Similarly, the use of biomass fuels within a house increases the risks of recurrent respiratory tract infections, poor lung growth, and the development of asthma and recently has been shown to have led to the development of chronic obstructive pulmonary disease in individuals as young as 35 years ^45– 47^. The use of natural gas as a source of cooking fuel increases the levels of nitrogen dioxide in the household air and this has been associated with an increased prevalence of viral infections of the upper respiratory tract, more so in children ^48^. A similar increase in respiratory ailments has been reported in children exposed to cigarette smoke ^49^. The burning of mosquito coils has been shown to be associated with increased prevalence of asthma, allergic rhinitis, eczemas, allergic conjunctivitis, and tuberculosis.

A Spanish cohort study reported an increased prevalence of hospitalizations for sickle cell disease ^50^. The exposure to the household air pollutants as discussed previously leads to exaggerated oxidative stress in patients with sickle cell disease. This causes endothelial damage, resulting in increased hemolysis and hypercoagulability. This also leads to reduced nitric oxide bioavailability and triggers vaso-occlusion, resulting in increased hospitalizations of patients with sickle cell disease in pain crises. Long-term exposures to PM _2.5_ and nitric oxide have been shown to significantly increase systolic blood pressure, mean arterial pressure, and pulse pressure ^51^. Increased levels of oxidative stress, as a result of exposure to household air pollution, are primarily responsible for these changes in blood pressure. However, particulate matter is also known to find its way into the systemic circulation, causing systemic effects. Moreover, household air pollutants lead to an imbalance within the autonomic nervous system with a predominant sympathetic response. This puts a person exposed to household air pollutants at risk of atherosclerosis, angina, ventricular hypertrophy, hypertension, pulmonary hypertension, ventricular conduction defects, and arrhythmias, leading to increased cardiovascular mortality and gross morbidity ^52^. Exposure to biomass fuel smoke has been associated with an acute increase in systolic blood pressure ^53^. The burning of incense sticks has been shown to increase the risks of cardiovascular mortality and stroke mortality by 1.12- and 1.19-fold, respectively. Household air pollutant levels on account of stir-frying as a mode of cooking, especially in Chinese cuisines ^8^, have been shown to decrease heart rate variability by 4.5%. Similarly, the use of detergents and cleaning products and the burning of incense are linked to decreased heart rate variability of 3.4% and 2.3%, respectively ^9^.

Furthermore, the oxidative stress caused by exposure to household air pollutants aggravates insulin resistance, which is implicated in type 2 diabetes mellitus ^54^. Exposure to third-hand smoke leads to similar effects on insulin resistance and development of non-obese type II diabetes mellitus ^55^. These patients with diabetes are more prone to the detrimental effects of PM _2.5_ and lower-sized particulate matter, leading to increased serum levels of inflammatory biomarkers, coagulation factors, and vasoconstrictor mediators, predisposing them to increased cardiovascular morbidity and subsequently amplified mortality. Obesogens, as discussed previously, affect the endocrine system, increasing the risk of metabolic syndrome and diabetes as well as cardiovascular risks ^29^.

Household air pollutants are also implicated in cognitive and judgmental skills ^56^. Cognition of older adults tends to be impaired up to 1.5 times with exposures to higher concentrations of particulate matter. The suggested pathway of cognitive impairment is through oxidative stress and activation of pro-inflammatory pathways. Particulate matter of 2.5 microns or less has been shown to cause depressive responses and impaired spatial learning and memory in animal studies. These pollutants were also found to increase hippocampal pro-inflammatory cytokine expression and change neuronal morphology ^57^. Significant gene-environmental interactions have been observed in individuals exposed to household air pollution, which increasingly predisposes them to developing lung cancers ^58, 59^. The use of coal, which produces a lot of smoke, for cooking and heating within the house has been implicated in an increased risk of developing small cell lung carcinoma, and this is attributed to mutations in over 68 genes ^60^. A group from Nepal has shown that exposure to biomass fuel led to a 1.7-fold increased risk of developing lung cancers ^61^. It is already a well-known fact that tobacco smoke increases the risk of developing lung cancers. Incense burning inside churches results in a 25- to 30-fold higher risk of oxidative DNA adducts than tobacco smoke particles ^22^.

## Interventions

### Creating awareness

Major interventional strategies need to be implemented at various levels to help curb this growing menace of household air pollution and its detrimental effects on health. The medical profession is attempting to improve the situation through increased research on the effects of household air pollution on health. Spreading this research through continued medical education will help disseminate this new knowledge among practitioners, increasing awareness about household air pollution and its health effects. Consolidating nationwide data by all countries will help generate regional data regarding household air pollution as different regions have varied causes and subsequently diverse strategies to implement changes to reduce this growing menace. Governments must formulate policies to bring about positive changes through behavioral interventions and cost-effective methods to improve fuels used for cooking and heating. For example, developed countries have now placed a ban on PentaPBDEs used in fire-retardant foam-containing furniture and electronics. This has helped reduce the levels of PentaPBDEs in their houses. The media can create tremendous public impact and aid the government and the medical profession in creating awareness about household air pollution among the general public.

### Remedial measures

The use of adulterated fuels and biomass for cooking and heating is one of the most important contributors to household air pollution. Advocacy to use cleaner fuels will assist in curbing this menace to a limited extent ^62^. One of the major deterring factors towards using cleaner cooking fuels is the economic burden of cleaner fuels. Improved cook stoves have been widely advocated to help reduce the emission from poor-quality cooking fuels ^63^. Locally fabricated top-lit updraft stoves used in Uganda ^64^ have significantly increased the efficiency of wood used, producing less smoke and more efficient and faster cooking. They also end up producing charcoal. However, cost remains an issue. Other forms of improved cook stoves have also been implemented. Although the use of these improved cook stoves helps reduce the particulate matter produced, this load remains well above the WHO safety limits ^65^. An 85% reduced exposure to particulate matter is required to achieve a desired clinical effect. The use of cleaner fuels such as LPG would reduce the load of household air pollutants to a great extent. A study conducted in Sudan reported a significant reduction in the levels of particulate matter (51–80%) and CO (74–80%) in homes that used LPG versus those that used biomass fuel for cooking ^66^. Widespread adoption of natural gas as a source of fuel helped reduce infant mortality by 4% in Turkey ^67^. In fact, certain governments have requested that their affordable populations forego their subsidy on LPG in order to provide for the disadvantaged sections of their societies. However, implementation of widespread use of LPG requires a colossal effort from healthcare providers, healthcare policy makers, local governments, industry, and, most importantly, the general public. Economic barriers for the use of LPG need to be sorted out through government policies that are more liberal than the existing ones ^68^.

In cases where improved cooking fuels are not feasible, every attempt must be made to improve the ventilation in these houses. Poor ventilation has been associated with a 49% increase in the risk of lung cancers ^60^. Implementing building guidelines and enforcing them may provide a solution to ensuring better ventilation in homes. Improving ventilation and the use of cleaner fuels help in reducing the rate of lung function decline as well. Improving ventilation may need additional behavioral interventions. People need to be educated about the efficiency of cleaner fuels, the use of traditional cooking fuels outside the home, the use of elevated kitchen platforms to facilitate quicker exit of biomass fuel smoke, the use of a long-stemmed chimney, or the addition of windows or doors to the home. A Sri Lankan study ^69^ reported that households using a chimney along with traditional cook stoves had a PM _2.5_ level of about 70 μg/m ^3^ but that households using traditional cook stoves without a chimney had PM _2.5_ levels of about 310 μg/m ^3^. However, local traditional cultures pose a challenge to behavioral interventions. Traditional methods of cooking, hampered use of cooking processes with improved cook stoves, non-conformity of improved cook stoves to traditional cookware, changes in flavor of food cooked on improved cook stoves, and lack of familiarity with the type of fuel used in improved cook stoves are the most important reasons for failure to implement improved cook stoves. A noteworthy case of behavioral intervention was reported by the Timor community of Indonesia ^70^. This society had a custom of enclosing a newborn baby and its mother in a confined ill-ventilated traditional house called Ume Kbubu. The two are exposed to fumes from a wood-burning stove in the belief that it will improve the general health and well-being of the new mother and her baby. Behavioral intervention of reducing this period of confinement from 40 days to 4 days significantly reduced the effects of the wood-burning stove. Similarly, behavioral interventions can be implemented to avoid the build-up of particulate matter from the burning of incense sticks. Advocating the use of mosquito bed-nets and window nets instead of the burning of mosquito coils or using aerosol mosquito repellents can assist in reducing the burden of household air pollutants.

It is not sufficient to improve only ventilation in households. Highly energy-efficient homes have also been found to be associated with increased risks of developing asthma ^71^. This is attributed to increased dampness within such high energy-efficient houses. It is therefore important not just to ensure energy efficiency and changed building materials but also to provide a dry and warm environment with good ventilation to minimize indoor dampness.

Air purifiers and ionizers have also been suggested as remedial measures to curb indoor air pollution. The use of air purifiers for just a couple of hours has been shown to reduce PM _2.5_ concentrations by 57% and caused a reduction in serum levels of inflammatory markers, indicating good cardiovascular outcomes ^72, 73^. However, no such statistical improvements have been shown in respiratory outcomes.

Using indoor plants, wet-mopping floors, avoiding the use of heavy upholstery, and refraining from smoking within confined home environments can further facilitate a reduction in household air pollutants. There are several other remedial measures suggested to reduce the health effects of household air pollution. However, these are beyond the scope of this review.

## Conclusions

Although a lot has been done in the arena of household air pollution, there is still room for further understanding the newer sources of indoor air pollution. Given the knowledge we have regarding household air pollution, long-term measures to curb its health effects have remained grossly insufficient. Stringent implementation of WHO guidelines on indoor air quality and a combined effort from the healthcare profession, industry, and healthcare policy makers can reinforce ways to curb household air pollution and, to an extent, limit its effects on health.
